# Behavioral Measures of Cochlear Gain Reduction Depend on Precursor Frequency, Bandwidth, and Level

**DOI:** 10.3389/fnins.2021.716689

**Published:** 2021-10-04

**Authors:** Kristina DeRoy Milvae, Elizabeth A. Strickland

**Affiliations:** Department of Speech, Language, and Hearing Sciences, Purdue University, West Lafayette, IN, United States

**Keywords:** cochlear gain reduction, forward masking, medial olivocochlear reflex, elicitor bandwidth, frequency selectivity, psychoacoustics

## Abstract

Sensory systems adjust to the environment to maintain sensitivity to change. In the auditory system, the medial olivocochlear reflex (MOCR) is a known physiological mechanism capable of such adjustment. The MOCR provides efferent feedback between the brainstem and cochlea, reducing cochlear gain in response to sound. The perceptual effects of the MOCR are not well understood, such as how gain reduction depends on elicitor characteristics in human listeners. Physiological and behavioral data suggest that ipsilateral MOCR tuning is only slightly broader than it is for afferent fibers, and that the fibers feed back to the frequency region of the cochlea that stimulated them. However, some otoacoustic emission (OAE) data suggest that noise is a more effective elicitor than would be consistent with sharp tuning, and that a broad region of the cochlea may be involved in elicitation. If the elicitor is processed in a cochlear channel centered at the signal frequency, the growth of gain reduction with elicitor level would be expected to depend on the frequency content of the elicitor. In the current study, the effects of the frequency content and level of a preceding sound (called a precursor) on signal threshold was examined. The results show that signal threshold increased with increasing precursor level at a shallower slope for a tonal precursor at the signal frequency than for a tonal precursor nearly an octave below the signal frequency. A broadband noise was only slightly more effective than a tone at the signal frequency, with a relatively shallow slope similar to that of the tonal precursor at the signal frequency. Overall, these results suggest that the excitation at the signal cochlear place, regardless of elicitor frequency, determines the magnitude of ipsilateral cochlear gain reduction, and that it increases with elicitor level.

## Introduction

An impressive feat that the human auditory system achieves is the ability to hear sounds that range from low to extremely high intensities. Most neurons in the auditory system respond sensitively to changes over a dynamic range of 30–40 dB, yet we are able to hear over a dynamic range of approximately 120 dB (Viemeister, 1988). This discrepancy between the dynamic range of nerve fibers and the dynamic range of hearing is referred to as the “dynamic range problem” (Evans, 1981; Viemeister, 1988). One way that the auditory system may overcome the dynamic range problem is by adapting its dynamic range based on the environment. Greater understanding of the adaptive nature of the auditory system has the potential to inform future treatments for hearing loss.

Efferent projections along the entire auditory pathway provide a possible means to adjust the dynamic range. A specific known physiological mechanism that is consistent with this function is the medial olivocochlear reflex (MOCR). The MOCR is an efferent pathway between the brainstem and cochlear outer hair cells that is elicited by sound and acts to decrease cochlear gain, with an onset delay of approximately 25 ms (James et al., 2005; Backus and Guinan, 2006). This gain reduction has been well documented physiologically in neural responses (Winslow and Sachs, 1987; Guinan and Gifford, 1988) and basilar membrane responses (Cooper and Guinan, 2003) in animal models, and in otoacoustic emission (OAE) responses (Backus and Guinan, 2006; Lilaonitkul and Guinan, 2009b) in humans. The MOCR is a bilateral reflex, with evidence suggesting that the ipsilateral pathway, where gain reduction is elicited by preceding sound in the same ear, may be stronger (Lilaonitkul and Guinan, 2012; but see Guinan et al., 2003). This makes the ipsilateral evoked response of interest and the focus of this paper.

The ipsilateral MOCR is elicited by preceding sound, but the frequency of the elicitor affects the magnitude of cochlear gain reduction. Neural measurements in cats have shown that olivocochlear bundle (OCB) fibers have tuning curves that are on average slightly broader than auditory nerve tuning curves and that the feedback loop is frequency-specific, such that preceding sound leads to larger reductions in gain near the cochlear place associated with that frequency (Liberman and Brown, 1986). Bonfils and Puel (1987) examined frequency selectivity of the MOCR by measuring forward masking of compound action potentials (CAPs), the synchronized response of the auditory nerve, in anesthetized guinea pigs to tone pips. These measurements were made with an intact and sectioned crossed (ipsilateral) OCB. Sectioning the crossed OCB caused a decrease in forward masking that occurred when the masker-onset to probe-onset was 40 ms, but not when that same duration was reduced to 30 ms. This suggests efferent contributions to forward masking that occur with a time delay between 30 and 40 ms. Functional tuning curves derived from the decrease in masking were relatively sharp (Q_10_ of 6.6) and centered on the probe frequency, suggesting again that the ipsilateral pathway is elicited in a frequency-specific way and that tuning is similar to that of afferent fibers (Q_10_ of 5–7.3; Bonfils et al., 1986). Similarly, tuning of ipsilateral MOCR effects is sharp when measured with stimulus frequency otoacoustic emissions (SFOAEs) in humans. In SFOAE measurements, the effects of preceding sound may be measured as the combined change in magnitude and phase of the SFOAE, or with magnitude and phase separated. It is not clear what measure is most relevant for the effects of the MOCR on perception. Tuning curves derived from ipsilateral elicitors, with magnitude and phase combined, showed sharp tuning for narrowband or tonal elicitors, with a tip near the probe frequency (Lilaonitkul and Guinan, 2009b). When magnitude and phase were separated, tuning for equal-input elicitors was sharp for magnitude, and more broadly distributed for phase (Lilaonitkul and Guinan, 2012).

In summary, both neural and SFOAE tuning data suggest that ipsilateral elicitation of the MOCR at a cochlear place is primarily driven by energy entering the auditory filter at that cochlear place. However, bandwidth effects have also been measured using SFOAEs that challenge this conclusion. MOCR effects increase with elicitor bandwidth and fixed overall level in a way not explained by additional excitation in the tails of the auditory filter, suggesting that there is integration of elicitation across almost the entire cochlea (Lilaonitkul and Guinan, 2009a) and that broadband noise stimuli are stronger elicitors of cochlear gain reduction than narrowband stimuli (e.g., Guinan, 2018). It is not clear if this bandwidth effect reflects a true difference between the MOCR in human and animal models, or if anesthesia or measurement techniques have led to these differences. Psychoacoustic methods provide an alternative approach to study decreases in cochlear gain in humans which may be due to the MOCR; behavioral measures could provide additional evidence for or against integration of elicitation with wider bandwidths.

**FIGURE 1:**
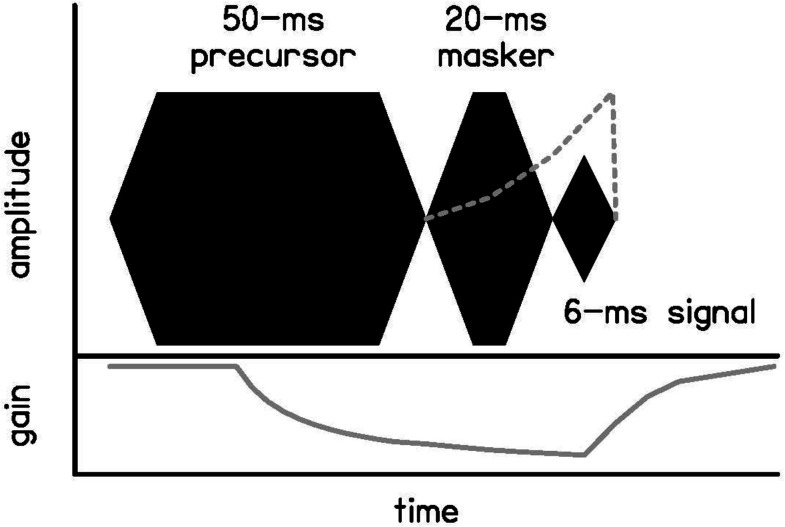
Schematic of the temporal masking paradigm used in this experiment, including a 50-ms precursor, 20-ms masker, and 6-ms signal. The precursor or masker is removed in some experiments, but the temporal relationships are not changed. The frequency content of the precursors and maskers also vary across experiments, but the signal is always presented at 4 kHz. The gray dotted line shows a schematic of the timecourse of forward masking due to neural excitation. The gray solid line shows a schematic of the timecourse of forward masking due to cochlear gain reduction with a precursor present.

Forward masking is a psychoacoustic method to explore cochlear gain reduction with eliciting preceding sound, called a precursor (Krull and Strickland, 2008; Jennings et al., 2009; Roverud and Strickland, 2014; Yasin et al., 2014; DeRoy Milvae and Strickland, 2018). Experimental design can be tailored to the time course of activation of the MOCR to estimate cochlear gain reduction with forward masking (e.g., Yasin et al., 2014; DeRoy Milvae and Strickland, 2018). With this approach (see example paradigm used in this experiment in Figure 1), the frequency content of the precursor can be varied to examine how frequency content of the elicitor affects gain reduction. Robust gain reduction has been measured with tonal (Jennings et al., 2009; Roverud and Strickland, 2014) and broadband noise (Yasin et al., 2014; DeRoy Milvae and Strickland, 2018) precursors, but comparisons have not yet been made within-subject to examine if the broadband noise precursors are more effective elicitors of cochlear gain reduction.

**FIGURE 2:**
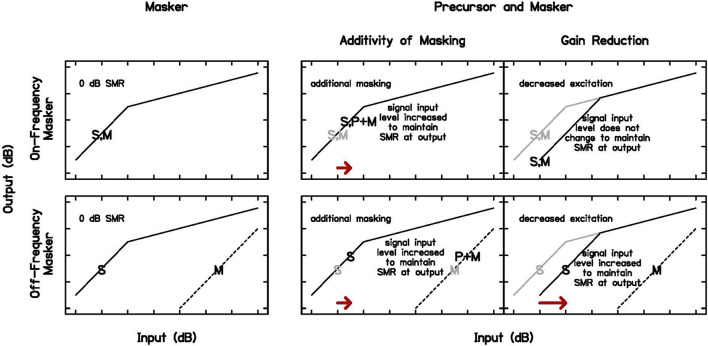
Schematic of cochlear input-output functions and threshold predictions in Experiment 1. Signal threshold (S) occurs at a criterion signal-to-masker ratio (SMR), in this case 0 dB (first column) for an on-frequency (top row) and off-frequency (bottom row) masker (M). With the addition of a precursor (P), predictions differ for forward masking due to additivity of masking or gain reduction. With additivity of masking (second column), a similar shift in signal threshold is expected when the same precursor is presented with equally effective on- and off-frequency maskers (arrows in second column). With gain reduction (third column), a larger shift in signal threshold is expected in the off-frequency case, since the masker is not affected by gain reduction at the signal frequency place (arrow in third column). The input-output functions, S, and M from the first column are repeated in gray in the second and third columns to illustrate the predicted changes with the introduction of a precursor.

However, cochlear gain reduction is not the only mechanism for forward masking. Neural excitation also plays a role in forward masking (see dotted line in Figure 1), and models based on this mechanism suggest additivity of masking, meaning that once compression is applied, the intensities of maskers add in their impact on the threshold of a closely following sound (Penner and Shiffrin, 1980; Oxenham and Moore, 1994; Plack et al., 2006). These models assume a static cochlear input-output function, but cochlear gain reduction occurs over time, affecting the cochlear non-linearity (Krull and Strickland, 2008; Roverud and Strickland, 2010). Previous work has shown that models including gain reduction fit data as well or better than those modeled with a static cochlear non-linearity (Jennings and Strickland, 2012; Roverud and Strickland, 2014). In one paradigm with a noise precursor, on-frequency masker, and 4-kHz signal, the signal level was fixed at 15–20 dB SL (sensation level) and masker threshold was measured for a range of precursor levels. The masker level had to be increased to effectively mask the signal with a precursor, more consistent with forward masking due to gain reduction than additivity of masking (Strickland et al., 2018). In this experiment, additivity of masking and gain reduction will again be compared, to establish that the forward masking in this experiment is more consistent with cochlear gain reduction. The paradigm to test this and the predicted results are shown in Figure 2. The Power Spectrum Model of masking is used in these predictions, such that detection occurs at a constant effective signal-to-masker ratio at the output of a single auditory filter at the signal frequency [for a review, see Jennings (2021)]. As in a similar paradigm at a lower frequency (DeRoy Milvae and Strickland, 2018), on- and off-frequency maskers will be obtained that elicit the same signal threshold (column 1 of Figure 2). An on-frequency precursor will then be added to each condition with the same temporal paradigm shown in Figure 1. Predictions are in the second two columns of Figure 2, for additivity of masking and gain reduction, respectively. If the additional masking is additive and does not change the cochlear non-linearity, a similar shift in threshold is expected with the addition of the precursor, not dependent on the frequency of the masker (arrows in column 2 of Figure 2). However, if the additional masking is related to cochlear gain reduction, no change in threshold is expected with an on-frequency masker, since the signal and masker are on the same function and are equally affected, but a large shift in threshold is expected with an off-frequency masker, since the signal is affected by the gain reduction and the masker is not (Cooper and Guinan, 2006; arrow in column 3 of Figure 2).

If the masking associated with the precursor is more consistent with cochlear gain reduction, the effects of precursor frequency content can be explored and interpreted in terms of gain reduction. In the case of tonal elicitors, it was hypothesized that gain reduction would occur in a frequency-specific way, as observed with tonal elicitors in previous physiological studies in both animal models and humans (Liberman and Brown, 1986; Bonfils and Puel, 1987; Lilaonitkul and Guinan, 2009b, 2012). In this case, an on-frequency precursor should be a more effective masker than an off-frequency precursor at the same level. Examination of forward masking with increasing precursor level also provides further evidence about tuning; because an on-frequency precursor grows compressively in the auditory channel at the signal frequency place, gain reduction should increase at a slower rate than 1 dB/dB with increasing precursor level. Because an off-frequency precursor should grow linearly in the auditory channel at the signal place, gain reduction should increase at a rate of approximately 1 dB/dB with increasing precursor level. Support for these hypotheses also comes from previous modeling of forward masking data. Modeling off-frequency-elicited gain reduction with level increasing with a slope of 1 dB/dB and on-frequency-elicited gain reduction with level with a shallower slope has predicted forward-masking data well (Roverud and Strickland, 2014). In addition, on- and off-frequency forward masking has been measured previously by Oxenham and Plack (2000), but not interpreted with consideration of cochlear gain reduction.

In the case of broadband noise elicitors, it was hypothesized that they would be more effective elicitors of cochlear gain reduction than tones, as observed in human SFOAE data (Lilaonitkul and Guinan, 2009a). To compare gain reduction with tones and noises, masking at the level of the noise entering an equivalent rectangular bandwidth (ERB; Glasberg and Moore, 1990), an estimated cochlear filter, will be compared to masking at the level of the tonal precursors. Greater masking with the noise would suggest integration across frequency to elicit gain reduction. If, however, the masking with the noise is similar to an on-frequency tone, it would suggest that integration across frequency does not take place and instead that ipsilateral cochlear gain reduction has similar tuning to that seen with afferent nerve fibers.

In this experiment, estimates of cochlear input-output functions were measured for individual participants using a forward masking technique. We hypothesized that shifts in input-output functions with preceding sound at 4 kHz are more consistent with cochlear gain reduction than additivity of masking, as observed previously with a similar paradigm at 1 kHz (DeRoy Milvae and Strickland, 2018). Cochlear gain reduction was examined as a function of the level and frequency content of preceding sound in an effort to examine how the peripheral auditory system remains sensitive across a wide range of input signals, and to examine how elicitation of cochlear gain reduction is tuned. We hypothesized that gain reduction would increase with precursor level, but the slope of increasing gain reduction with increasing precursor level would be shallower with an on-frequency precursor than with an off-frequency precursor, due to cochlear compression of the precursor at the signal place. This would suggest that gain reduction from an ipsilateral elicitor is driven by excitation in an auditory filter at or near the signal frequency, like other forms of forward masking. With a broadband noise precursor, we hypothesized that stronger gain reduction would be elicited than seen with tonal stimuli, as seen with SFOAE measurements in humans. The outcome of this research is an estimate of cochlear gain reduction in decibels, obtained through perceptual measures in humans.

## Experiment 1: Forward Masking With a Precursor Is More Consistent With Cochlear Gain Reduction Than Additivity of Masking

Growth-of-masking (GOM) functions were measured to obtain an estimate of each participant’s cochlear input-output function (Oxenham and Plack, 1997; Plack and Oxenham, 1998) with and without preceding stimulation, a precursor (Krull and Strickland, 2008; Jennings et al., 2009; Roverud and Strickland, 2010) under our temporal paradigm (see Figure 1). The additional masking with preceding sound could be interpreted as a decrease in cochlear gain, but there are other possible explanations, such as masking due to neural excitation, which predicts additivity of masking given a correction for peripheral compression (Penner and Shiffrin, 1980; Oxenham and Moore, 1994, 1995). A gain reduction hypothesis was tested against additivity of masking using on- and off-frequency forward maskers that resulted in the same signal threshold, making them equally effective maskers of the signal. When the same precursor is added to each condition, additivity of forward masking predicts a similar shift in threshold, regardless of masker frequency. However, gain reduction predicts that the addition of a precursor before an off-frequency masker will lead to a larger shift in threshold (see Figure 2). Because an off-frequency masker is processed linearly at the signal place at basal frequencies (Cooper and Guinan, 2006), its gain is not reduced by preceding on-frequency sound, and it is predicted to be a more effective forward masker.

### Methods

#### Participants

Seven young adults (P1–P7) between the ages of 19 and 26 years (median: 21 years) participated in this experiment. All were female except for P5, who was male. All participants had normal audiometric thresholds (15 dB HL or less) at octave frequencies from 0.25 to 8 kHz and present distortion product otoacoustic emissions from 1.5 to 10 kHz. Some participants did not take part in all experiments.

#### Stimuli

##### Growth of Masking

Two types of GOM functions were measured for each participant in a forward masking paradigm to estimate the cochlear input-output function at full gain (without reduction in cochlear gain associated with prior sound stimulation) and reduced gain. For the full-gain GOM function, stimuli consisted of a 20-ms, 2.4-kHz tonal masker (including 5-ms cos^2^ onset and offset ramps) followed by a 6-ms, 4-kHz tonal signal (including 3-ms cos^2^ onset and offset ramps) with no time delay between masker and signal. As in previous studies (e.g., DeRoy Milvae and Strickland, 2018), this masker and signal duration were chosen to be near the estimated onset delay of 20–25 ms for the MOCR (James et al., 2005; Backus and Guinan, 2006), so that there is very little MOCR activation, if any, in this condition. Masker level was fixed between 30 and 95 dB SPL in order to trace out a GOM function for each individual. Signal level was varied to determine the signal level at masking threshold.

A second GOM function at reduced gain was measured for each individual using the same masker and signal, but with the addition of preceding sound before the masker, called a precursor (see Figure 1 for temporal paradigm). This function was measured with a 50-ms, 40 dB SPL, 4-kHz tonal precursor (including 5-ms cos^2^ onset and offset ramps) presented prior to the masker and signal. The precursor duration was 50 ms, as this has been found to be the most effective duration for an on-frequency precursor to shift threshold given this temporal paradigm (Roverud and Strickland, 2014). A level of 40 dB SPL for a tonal on-frequency precursor has been found to produce robust gain reduction in previous studies (Roverud and Strickland, 2010; Jennings and Strickland, 2012).

In addition to the GOM functions, gain reduction was estimated by comparing the signal threshold in quiet to the signal threshold preceded by the precursor and no masker, with a 20-ms silent gap between precursor and signal (in place of the masker). This estimate has shown to be consistent with gain reduction estimates measured with a masker present (DeRoy Milvae and Strickland, 2018; DeRoy Milvae et al., 2021) for listeners with normal thresholds in quiet.

##### Equally Effective Maskers

On-frequency maskers were identified that were equally effective (produced the same signal threshold) as off-frequency maskers used to measure GOM functions. The 6-ms, 4-kHz signal (including 3-ms cos^2^ onset and offset ramps) was fixed at the threshold level obtained when it was preceded by a 20-ms, 2.4-kHz masker (including 5-ms cos^2^ onset and offset ramps). The level of a 20-ms, 4-kHz masker (including 5-ms cos^2^ onset and offset ramps) was then varied to measure threshold and find the lowest masker level where the signal could be detected. This level was then confirmed to produce the same signal threshold as the off-frequency masker by fixing the masker level and varying the signal level. This was done for two points on the lower leg of the GOM function for each participant, although an effect of masker frequency with the addition of a precursor was expected as long as the point chosen was not affected by compression.

To examine whether shifts in forward masking with a precursor were more consistent with gain reduction than additivity of masking, an identical precursor was presented before the two equally effective maskers and signal threshold was measured in each condition (measurements from the GOM function used for the off-frequency conditions). Additivity of masking predicts that adding a 50-ms, 40 dB SPL, 4-kHz precursor (including 5-ms cos^2^ onset and offset ramps) before the on-frequency masker and off-frequency masker that produce the same signal threshold should cause an identical shift in threshold (see column 2 of Figure 2). This method does not rely on the measurement of the input-output function for interpretation. It was hypothesized that a larger shift in signal threshold would be seen for the off-frequency condition, more consistent with precursor masking related to cochlear gain reduction (see column 3 of Figure 2).

#### Procedure

The experiment took place in a double-walled sound-attenuating booth (IAC, Bronx, NY, United States). Tucker–Davis Technologies (TDT, Alachua, FL, United States) hardware was used. Stimuli were digitally generated at a sampling rate of 25 kHz. They were then sent to four separate digital-to-analog channels (TDT DA3-4, 16-bit), low pass filtered at 10 kHz (TDT FT5 and FT6-2), mixed (TDT SM3), buffered (TDT HB6), and output to the participant’s right ear *via* an ER-2 (Etymotic Research, Inc., Elk Grove Village, IL, United States) insert earphone. This insert earphone has a flat frequency response at the eardrum for frequencies from 0.25 to 8 kHz. Participants wore both the left and right earphones, even though sound was not presented to the left ear, to reduce interference from ambient noise.

Participants performed a three-interval forced choice task. Intervals were separated by 500 ms of silence and participants indicated the interval containing the signal by pressing a key. Visual indicators were used to identify the intervals and feedback was given to indicate the veracity of the participant’s choice. The signal level was adjusted while the masker level was held constant to approximate a detection threshold of 70.7% correct on the psychometric function (Levitt, 1971) for the range of masker levels tested. To determine the on-frequency masker levels needed to elicit a similar signal threshold as off-frequency maskers, the masker level was adjusted while the signal level was held constant. Participants completed 2–5 h of training on GOM tasks to control for learning effects and 1–3 h of training with on-frequency masker conditions for the equally effective maskers task. Less training was needed on this task because participants were already familiar with the general forward masking task. Two runs for each condition collected on the final day of participation are included in the experimental data. However, on-frequency masked thresholds of P2 continued to show high variability after training. For this reason, more than two estimates of each threshold were attempted for this participant, with an average of 3.5 threshold estimates measured per condition that did not have to be removed from experimental data due to high standard deviations. Off-frequency conditions were also repeated for this subject instead of using the measurements from the GOM function, so that measurements with equally effective maskers were collected at a similar point in time for this highly variable listener. In addition, an experimenter error led to three thresholds collected for P5 in the 65 dB SPL off-frequency masker and precursor condition (reduced gain GOM function and off-frequency equally effective masker condition with a precursor), but this additional threshold was similar to the first two measured and was not believed to influence the results.

During each masked trial, high pass noise was presented to limit off-frequency listening (Nelson et al., 2001). It began 50 ms before the first stimulus and ended 50 ms after the signal. The noise was presented at a spectrum level 50 dB below the signal level (varying adaptively with the signal level), and had 5-ms cos^2^ onset and offset ramps and a bandwidth of 4.8–8.0 kHz. Because P2 demonstrated difficulty with the tasks when the high pass noise was present, resulting in inconsistent thresholds across trials, the noise was removed during testing for this participant.

Each run consisted of 50 trials. The step size was 5 dB before the second reversal in signal (or masker) level, and then the step size decreased to 2 dB. Runs were excluded if the standard deviation was greater than 5 dB for one or two final runs or if less than six reversals were present. The final even number of reversals at the 2-dB step size were averaged to estimate threshold for each run.

### Results

#### Growth of Masking

**FIGURE 3:**
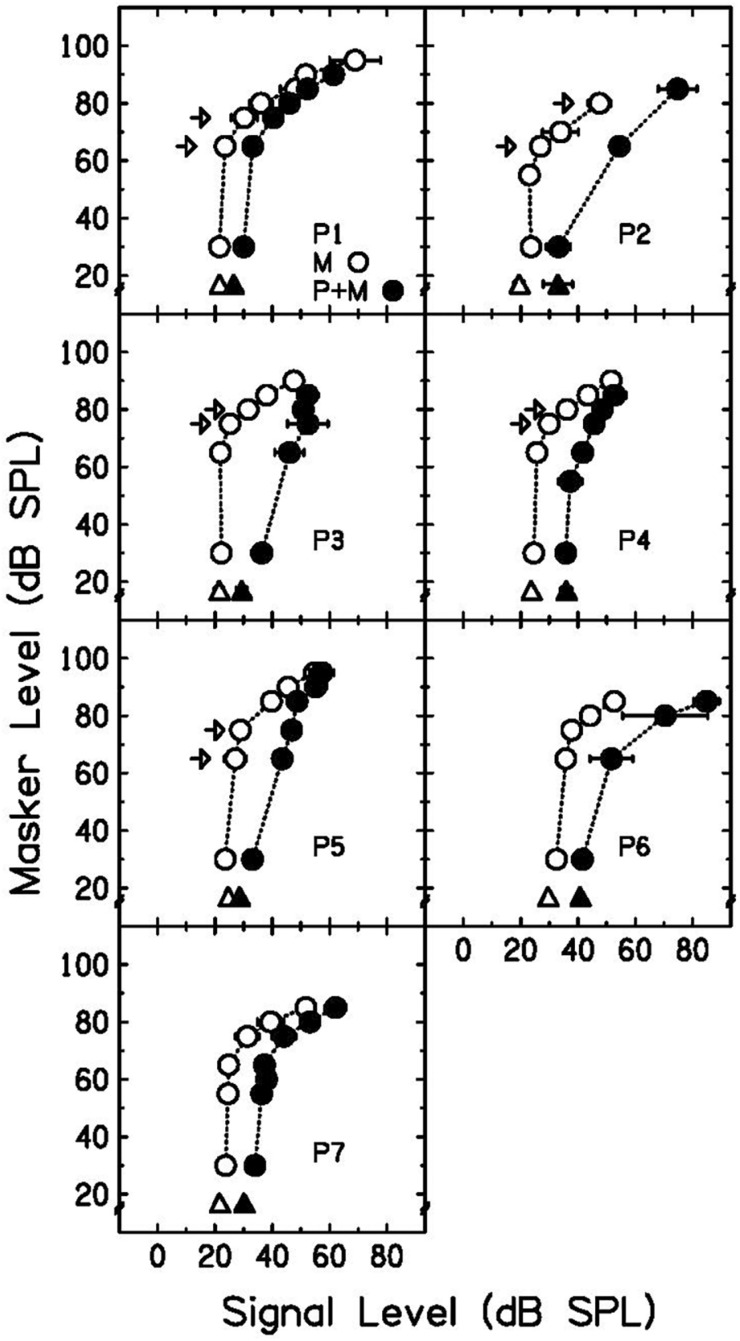
Individual GOM functions and masker-absent gain reduction estimates. Signal thresholds for the masker-alone condition are plotted as open circles and signal thresholds with the addition of a precursor are plotted as filled circles. Signal threshold without a preceding masker is plotted as an open triangle, and signal threshold with a precursor and 20-ms delay is plotted as a filled triangle. The difference between the triangles is the masker-absent gain reduction estimate. Arrows indicate the off-frequency masker levels used in the equally-effective-masker conditions. Error bars represent one standard deviation.

Growth-of-masking functions without a precursor (open circles) and with a precursor (filled circles) are plotted in Figure 3. Open triangles represent the signal threshold when the signal is presented alone. Filled triangles represent the signal threshold when the precursor is present but there is no masker (20-ms gap of silence between precursor and signal). As shown in previous work (Krull and Strickland, 2008; Jennings et al., 2009; Roverud and Strickland, 2010), the precursor shifted the lower leg of the GOM function to higher signal levels (a rightward shift). This shift is consistent with a decrease in cochlear gain. P2 had a limited number of thresholds for the precursor condition because this participant’s runs often resulted in standard deviations that were above 5 dB, and those thresholds were not included. It was observed that the masker-absent gain reduction estimate (difference between open and filled triangles in Figure 3) was a reasonable estimate for the gain reduction observed by the shift in the GOM function, as shown previously (DeRoy Milvae and Strickland, 2018; DeRoy Milvae et al., 2021).

#### Equally Effective Maskers

**FIGURE 4:**
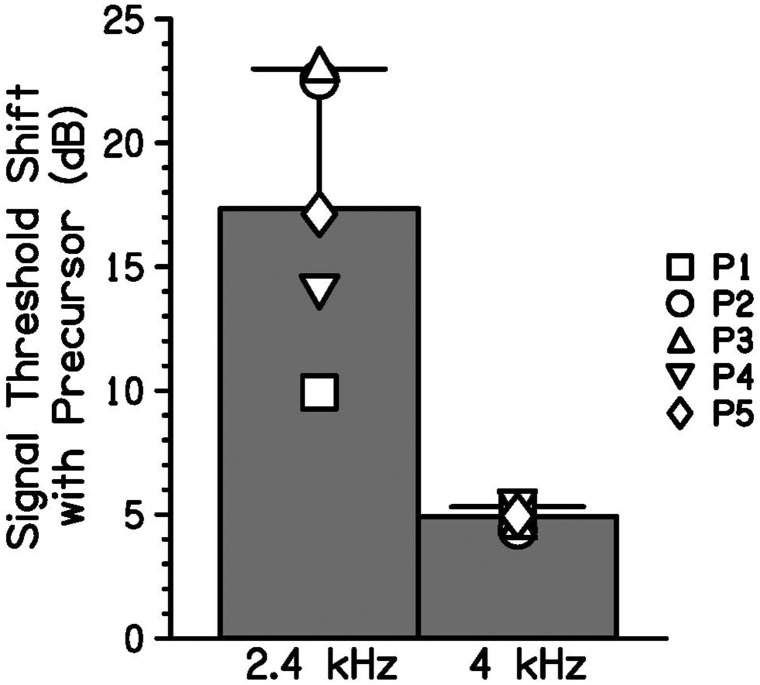
Bars indicate the group average increase in signal threshold with a precursor preceding equally effective off-frequency (2.4-kHz) and on-frequency (4-kHz) maskers. Signal threshold shift with a precursor was averaged for two matched signal levels for each participant (symbols). Error bars represent one standard deviation.

Individual signal thresholds are shown in Table 1 and average threshold shifts with the addition of a precursor at two masker frequencies are shown in Figure 4. As was shown in Figure 3, the precursor shifted signal thresholds to higher levels when the masker was 2.4 kHz. In the 4 kHz masker case, there was a much smaller shift in threshold. One-tailed *t*-tests (with a Holm-Bonferroni correction) were performed to test for significance that the threshold for the off-frequency condition with an added precursor was higher than that of the on-frequency condition with an added precursor at the individual level, and significant differences (*p* < 0.05) are noted by asterisks in Table 1. P1, P2, and P3 showed a significantly higher threshold for the off-frequency condition with an added precursor at one level of matched threshold, *t*(2) = 8.03, *p* = 0.046; *t*(6) = 8.43, *p* < 0.001; and *t*(2) = 5.23, *p* = 0.041; respectively. P5 showed this same effect at two levels of matched threshold. For a matched threshold of 27 dB SPL, *t*(3) = 6.98, *p* = 0.027, and for a matched threshold of 29 dB SPL, *t*(2) = 9.57, *p* = 0.043. Other participants showed a similar trend that did not reach significance. In addition, the data were averaged across participants by taking the average difference between the precursor condition and the masker-alone condition for each masker frequency (averaging the two levels for each participant). A one-tailed *t*-test was performed for these data and there was a significant difference between the average change in threshold for a 2.4-kHz masker and a 4-kHz masker when an identical precursor is added, *t*(8) = 4.91, *p* = 0.006.

**TABLE 1:** Individual data with equally effective maskers.

Participant	Masker level		Masked signal threshold		Masked signal threshold with 4-kHz precursor	
	2.4-kHz masker	4-kHz masker	2.4-kHz masker	4-kHz masker	2.4-kHz masker	4-kHz masker
P1	65	16	23.53 (1.60)	22.97 (0.20)	*33.17 (0.24)	27.67 (0.94)
	75	33	30.20 (4.53)	29.98 (1.21)	40.42 (3.24)	35.85 (0.68)
P2	65	21	28.65 (1.09)	33.85 (11.54)	*50.80 (2.90)	36.42 (1.89)
	80	45	45.41 (4.59)	51.68 (5.56)	68.36 (9.77)	57.89 (2.31)
P3	75	14	25.25 (1.06)	22.30 (0.42)	52.40 (7.07)	26.27 (0.09)
	80	30	31.61 (1.15)	29.72 (0.08)	*50.77 (2.27)	35.16 (0.79)
P4	75	28	29.93 (1.52)	30.76 (1.28)	45.60 (1.98)	39.24 (4.38)
	80	37	36.18 (1.44)	41.81 (3.37)	48.46 (0.94)	43.93 (3.21)
P5	65	21	27.12 (3.84)	26.08 (4.36)	*43.48 (2.17)	32.19 (0.04)
	75	27	28.89 (1.97)	29.78 (1.96)	*46.78 (1.96)	33.54 (0.05)

*Mean masked signal threshold (dB SPL) is shown for two masker levels chosen to elicit similar masked thresholds with both masker frequencies.*

*Masked signal threshold with the addition of the same precursor is shown for off- and on-frequency masker conditions.*

*One standard deviation is shown in parentheses.*

*Signal thresholds for off-frequency masker conditions with a precursor that were significantly higher than the corresponding on-frequency masker condition with a precursor are indicated with an asterisk.*

### Discussion

The shift in signal threshold with a precursor and no masker (difference between open and filled triangles in Figure 3) was demonstrated to be a reasonable estimate of gain reduction, as observed previously (Roverud and Strickland, 2010; DeRoy Milvae and Strickland, 2018). However, in some cases it was lower than that observed in the GOM function; for example, the masker-absent threshold shift was smaller than that with a masker present for P3. Lower estimates may be found with this method since the MOCR can reduce the spontaneous rate of auditory nerve fibers (Guinan and Gifford, 1988). Therefore, the masker-absent estimate of gain reduction may sometimes underestimate gain reduction.

With equally effective maskers that differed in frequency, a larger shift in threshold was induced for the off-frequency masker condition than for the on-frequency masker condition with the introduction of an on-frequency precursor (Figure 4). Since the change in threshold depended on masker frequency, the masking provided by the precursor was more consistent with gain reduction than neural excitation alone. Additivity of masking would predict a similar change in threshold, regardless of masker frequency. The current data show that when the effects of a precursor on an on-frequency and off-frequency masking condition are compared, the change in signal threshold is not easily explained by additivity of masking. This difference in threshold shift measured was consistent with gain reduction in that the precursor in both cases elicits gain reduction at the 4-kHz place, differentially affecting the on- and off-frequency maskers. Since the 2.4-kHz masker should have an approximately linear response at the 4-kHz place, it is not affected by the gain reduction elicited by the precursor and is thus a more effective masker than the 4-kHz masker in this condition. This leads to a greater shift in threshold for the off-frequency masker condition. Even with this differential effect, some change in threshold can be seen for the on-frequency masker. This effect is still consistent with gain reduction. It can occur if the gain is decreased enough that the signal becomes inaudible. Alternatively, residual additivity of masking, after accounting for gain reduction, could also explain the increase in threshold with an on-frequency masker.

This result is similar to that observed previously at 4 kHz (Jennings et al., 2009) and at a lower signal frequency (DeRoy Milvae and Strickland, 2018). A differential effect of a precursor on masking of a signal by on- and off-frequency maskers below the signal frequency has also been seen in studies in which the signal level was fixed and the masker level was varied to measure a psychoacoustic tuning curve or a temporal masking curve. In these cases, the addition of the precursor decreases the masker level needed to mask the signal for the off-frequency masker, but not for the on-frequency one. This has been seen with a contralateral precursor (Kawase et al., 2000; Fletcher et al., 2016) and an ipsilateral precursor (Jennings and Strickland, 2012).

Additional evidence supporting a gain reduction explanation comes from Roverud and Strickland (2014), a study exploring differences in forward masking with on- and off-frequency precursors. They measured the shift in threshold following an off-frequency masker produced by an on- or off-frequency precursor, as a function of precursor duration. For the 2.4-kHz precursor, threshold increased with precursor duration for durations up to 160 ms. For the 4-kHz precursor, however, threshold increased with precursor duration up to 50 ms, but then either plateaued or in some cases oscillated. This was modeled using a temporal window model combined with gain reduction elicited by the precursor. For an on-frequency precursor, the precursor itself was affected by gain reduction, and thus effectiveness fluctuated with duration. The off-frequency precursor was not affected by gain reduction within the signal channel, and thus effectiveness continued to grow with duration.

## Experiment 2: Signal Threshold With Increasing Level of Tonal Precursors

The results of Experiment 1 support the theory that a shift in signal threshold with a precursor reflects gain reduction. In that case, it is of interest to examine gain reduction as a function of precursor frequency and level, to examine the tuning of cochlear gain reduction elicitation. The results of previous studies suggest that gain reduction may increase at a slope of approximately 1 dB/dB of increasing precursor level for a masker well below the signal frequency (Oxenham and Plack, 2000; Roverud and Strickland, 2014), and increase at a shallower slope for a masker at the signal frequency (Plack and Oxenham, 1998; Oxenham and Plack, 2000; Roverud and Strickland, 2014). This experiment replicates and builds on aspects of the design of Oxenham and Plack (2000), and results are interpreted taking into account a gain reduction hypothesis.

### Methods

#### Participants

The same seven participants from Experiment 1 (P1–P7) between the ages of 19 and 26 years (median: 21 years) participated in this experiment.

#### Stimuli

A 50-ms, 2.4- or 4-kHz precursor (including 5-ms cos^2^ onset and offset ramps) was presented with a 20-ms silent gap before the signal, a 6-ms, 4-kHz tone (including 3-ms cos^2^ onset and offset ramps). Precursor levels were fixed between 20 and 90 dB SPL for the 4-kHz precursor (on-frequency) condition and between 60 and 95 dB SPL for the 2.4-kHz (off-frequency) condition in order to trace out changes in signal threshold for each individual.

#### Procedure

Equipment and procedures were identical to those of Experiment 1 with the following exceptions. Signal level was adjusted while the precursor level was held constant during measurements (the signal level was not held constant for any measurements in this experiment). Additionally, approximately 2–5 h of training were completed on these tasks to control for learning effects.

### Results

**FIGURE 5:**
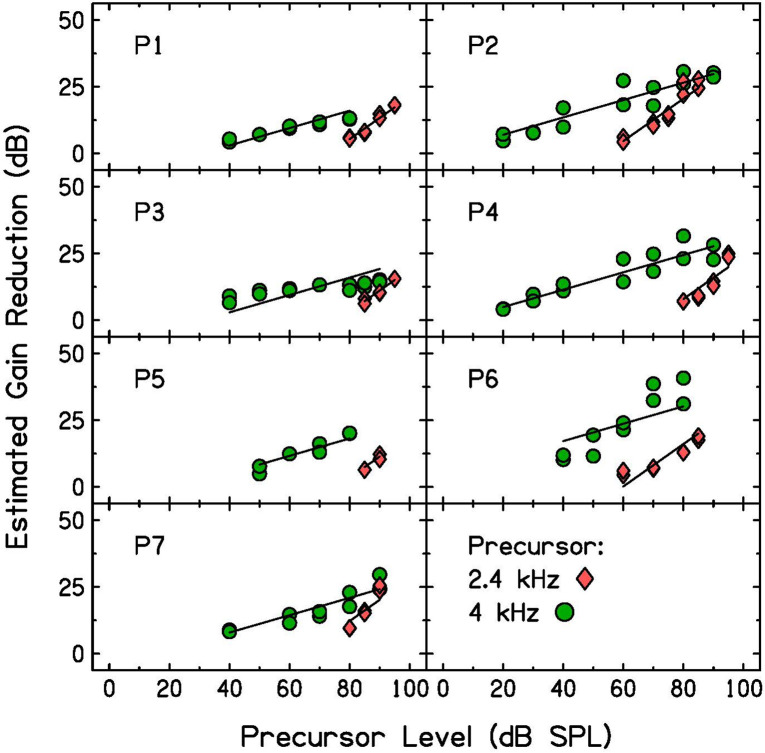
Individual estimated gain reduction with precursor level measured with 2.4-kHz (pink diamonds) and 4-kHz (green circles) precursors. Estimated gain reduction was calculated by subtracting quiet threshold for the signal from the signal threshold for each condition. The two data points measured for each precursor level and included in the analysis are plotted. Linear mixed-effects model fits (see Table 2 for model summary) are plotted as lines over the data.

Gain reduction was estimated by subtracting each participant’s threshold for the signal alone (quiet threshold, shown as open triangles in Figure 3) from their signal threshold with the precursor measured in this experiment. A shift in signal threshold due to the presence of the precursor was interpreted as estimated gain reduction. Estimated gain reduction is plotted as a function of precursor level in Figure 5. Gain reduction increased with precursor level more rapidly in the off-frequency masker condition (pink diamonds) than in the on-frequency masker condition (green circles).

**TABLE 2:** LMM summary describing the effects of precursor level and frequency on gain reduction estimates (dB).

	Estimated gain reduction (dB)			
Fixed effects	Estimate	*SE*	*t*	*p*
Intercept	–4.32	2.49	–1.74	0.11
**Level**	**0.33**	**0**.**02**	**16.03**	**<0.001**
**Frequency (2.4 > 4 kHz)**	−**49.47**	**6.27**	−**7.89**	**<0.001**
**Level × Frequency**	**0.47**	**0.07**	**6.37**	**<0.001**

**Random effects**	**Variance**	***SD***	**Correlations**	

By-participant intercepts	31.23	5.59		
By-participant frequency slopes	16.18	4.02	–0.02	
Residual	11.98	3.46		

*Significant fixed effects are shown in bold.*

*The *p-*values were calculated with a Satterthwaite approximation.*

To test whether the slope was significantly different with precursor frequency, a linear mixed-effects model (LMM) was used to fit this data set. All data points were included that provided over 4 dB of estimated gain reduction to remove floor effects that would affect the slope (the data shown in Figure 5). The dependent variable was estimated gain reduction (dB). Fixed effects included in the model were precursor frequency (a categorical variable of 2.4 or 4 kHz, with 4 kHz chosen as the reference level) and precursor level (a continuous variable), and the interaction between precursor frequency and level. A significant interaction would be interpreted as a significant difference in slope of the functions with precursor frequency. Random intercepts and slopes were included as random effects in the maximal model for precursor frequency by participant. Model testing was completed using R 4.0.3 (R Core Team, 2020) and the “buildmer” version 1.5 (Voeten, 2020) package. The buildmer function ordered effects using the likelihood-ratio test statistic (LRT), followed by backward-elimination model testing based on the significance of changes in log-likelihood. With this approach and the maximal model as input (Barr et al., 2013), a model was found that both converged and best fit the data (Matuschek et al., 2017; Voeten, 2020). The Satterthwaite approximation (Luke, 2017) was used to generate *p*-values. The model summary for the model of best fit is in Table 2. There is a significant interaction between precursor level and frequency, such that the slope is significantly shallower in the on-frequency precursor condition. The slope of the on-frequency precursor condition fit was 0.33, and the slope of the off-frequency precursor condition fit was 0.47 higher, with a slope of 0.80 (see Table 2). Thus, the slope was far shallower in the on-frequency precursor condition.

### Discussion

The slope of estimated gain reduction with increasing precursor level depended on precursor frequency (Figure 5 and Table 2). This finding is consistent with previous model assumptions. Roverud and Strickland (2014) used a gain reduction model based on the timecourse of the MOCR, followed by a temporal window model, to model forward masking with increased precursor duration. In the model, the input to the gain-reduction module was assumed to grow compressively for an on-frequency precursor, using the compression derived from each listener’s input-output function. For the off-frequency precursor, the input to the gain reduction module was assumed to grow linearly. With these assumptions, the data were fit well. The model incorporating gain reduction fit the data better than a model using only a temporal window. The results of the present study are consistent with those results, in that the growth of gain reduction with precursor level has a shallow slope with an on-frequency precursor, and has a more linear slope with an off-frequency precursor. This suggests physiologically that the precursor is processed at the signal place, such that on-frequency sound is affected by cochlear compression, and that the output of the cochlear non-linearity serves as a local input to cochlear gain reduction.

The estimated gain reduction measured in this experiment is very similar to forward masking measured by Oxenham and Plack (2000). Oxenham and Plack (2000) measured growth of forward masking with masker level for 0-, 10-, and 30-ms delays between a 200-ms masker (on- and off-frequency) and a 10-ms, 4-kHz signal. For on-frequency maskers, the slope of increased masking with increased masker level became shallower with longer delays between the masker and signal. For off-frequency maskers, delay did not affect the slope. Although Oxenham and Plack (2000) did not interpret their data in terms of cochlear gain reduction, gain reduction estimates can be made from the 30-ms delay data they presented by subtracting quiet threshold for each participant from the masked thresholds presented. On-frequency data show approximately 20–35 dB of maximum gain reduction for the participants and off-frequency data shows approximately 25–40 dB of maximum gain reduction for the participants. The present results had similar ranges of maximum gain reduction. Although Oxenham and Plack (2000) interpreted their results using a temporal window model (additivity of masking), this model did not predict the rollover (decrease in threshold) observed with increased masker duration. Similar rollover effects have been modeled well with gain reduction (Roverud and Strickland, 2014). Therefore, cochlear gain reduction is an alternative explanation for the Oxenham and Plack (2000) data.

The on- and off-frequency functions are consistent with the idea that excitation at the signal place, regardless of the frequency presented to the ear, leads to cochlear gain reduction which increases with level. Gain reduction increases at a slower rate if the precursor itself is compressed in the system, as is the case with an on-frequency precursor.

## Experiment 3: Signal Threshold With Increasing Level of Broadband Noise Precursors

There is evidence from otoacoustic emission data that stimuli with wider bandwidths are more effective elicitors of ipsilateral cochlear gain reduction (Lilaonitkul and Guinan, 2009b). It is believed that broadband noise is more efficient in eliciting the MOCR than stimuli with smaller bandwidths, such as narrowband noise or tones (Norman and Thornton, 1993; Maison et al., 2000; Guinan et al., 2003). In addition, many studies investigating contralateral MOCR activity in humans with otoacoustic emissions use 60-dB SPL broadband noise as an elicitor (e.g., Guinan et al., 2003; Backus and Guinan, 2006; Francis and Guinan, 2010). However, in psychoacoustic studies, both tones and noises have been used as elicitors, and have shown large amounts of gain reduction (Jennings et al., 2009; Roverud and Strickland, 2014; Yasin et al., 2014; DeRoy Milvae and Strickland, 2018). Therefore, it is also of interest to measure growth of gain reduction when the precursor is a broadband noise and compare to growth of gain reduction with pure tones, to examine if behavioral measures of cochlear gain reduction with a wideband stimulus are consistent with integration of gain reduction elicitation across the cochlea.

### Methods

#### Participants

P1–P5 from Experiments 1 and 2 returned to complete Experiment 3. However, P5 was removed from the study for the reasons described in the “Stimuli” section. The ages of P1–P4 range 20–26 years (median: 22 years).

#### Stimuli

Gain reduction was estimated as a function of precursor level, as in Experiment 2, but the precursor in this experiment was a broadband noise (BBN) rather than a pure tone. The BBN was 80–10,000 Hz wide and levels ranged from 24 to 64 dB overall RMS level. Higher levels were not tested to avoid confounding effects from the middle-ear muscle reflex, elicited at a lower level by noises than tones (e.g., Schairer et al., 2007). The BBN precursor was 50-ms in duration (including 5-ms cos^2^ onset and offset ramps) and was presented with a 20-ms gap before the 6-ms, 4-kHz (including 3-ms cos^2^ onset and offset ramps) signal. It was hypothesized that the increase in estimated gain reduction with precursor level would have a steeper slope than that of an on-frequency precursor if elicitation is integrated across cochlear place. Alternatively, if on-frequency energy of the noise dominates elicitation of gain reduction at the signal-frequency place, a similar slope was expected as seen with on-frequency tones.

In the MATLAB program, an error was present which led to the removal of data from P5. When generating the BBN, the program used a frozen noise for the two intervals without the signal and generated a second noise for the interval with the signal. Because of this, participants were able to listen for a change in the noise (which was not changing in level across presentations) rather than listen for the signal. P5 was the only participant to use this cue, leading to impossibly low thresholds. Because other participants’ thresholds were not impossibly low, it was assumed that they did not use the noise cue available and their data are presented, although some contribution of this cue cannot be entirely ruled out.

#### Procedure

Experiments took place in a sound-attenuated booth (IAC, Bronx, NY, United States). A custom program developed using MATLAB software (2011a, The Math Works, Natick, MA, United States) was used to present stimuli. Stimuli were generated in MATLAB *via* a Lynx TWO-B sound card (Lynx Studio Technology, Inc., Costa Mesa, CA, United States). They were then buffered (TDT HB6) and presented to a right ER-2 insert earphone.

The procedure was identical to that of Experiment 2 except for a difference in the criterion for number of trials in the adaptive procedure. The step size was 5 dB before the fourth reversal; it then decreased to a step size of 2 dB for the remainder of the trials. Trials continued until 12 reversals were completed, and the final eight reversals were averaged to establish threshold.

### Results

**FIGURE 6:**
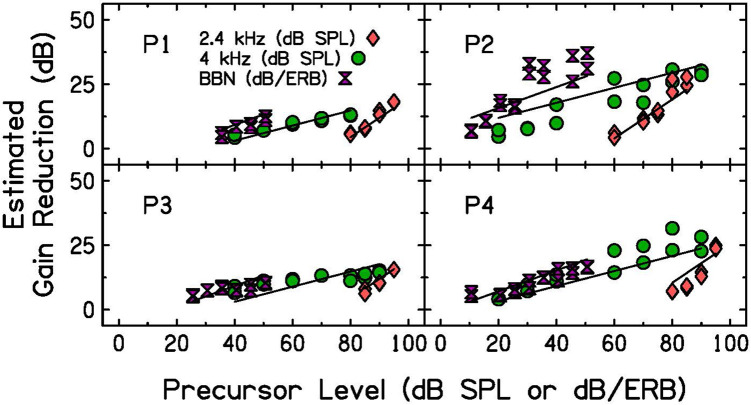
Individual estimated gain reduction with precursor level measured with BBN (purple hourglasses), 2.4-kHz (pink diamonds, identical to data presented in Figure 5) and 4-kHz (green circles, identical to data presented in Figure 5) precursors. Precursor level is plotted in dB per equivalent rectangular bandwidth (ERB) for the noise precursors. Estimated gain reduction is calculated by subtracting quiet threshold for the signal from the signal threshold for each condition. LMM fits (see Table 3 for model summary) are plotted as lines over the data.

Estimated gain reduction with BBN precursors is plotted as a function of precursor level in Figure 6 (purple hourglass symbols), with the tonal precursor data from Experiment 2. Gain reduction estimates were calculated in the same way as in Experiment 2; quiet threshold for the signal alone was subtracted from the threshold for the signal with the BBN precursor at each level. Level of the BBN is plotted as the decibel level per equivalent rectangular bandwidth (dB/ERB) of the noise. This calculation was done to approximate the level of the sound entering the cochlear filter centered at the signal frequency, 4 kHz. The equation used to calculate the ERB (Glasberg and Moore, 1990) is shown below, where F is the frequency of the signal in kHz and ERB is the filter bandwidth at that frequency.


(1) E⁢R⁢B=24.7⁢(4.37⁢F+1)

**TABLE 3:** LMM summary describing the effects of precursor level and frequency content on gain reduction estimates (dB).

	Estimated gain reduction (dB)			
Fixed effects	Estimate	*SE*	*t*	*p*
Intercept	–3.40	3.86	–0.88	0.42
**Level**	**0.29**	**0**.**03**	**11.38**	**<0.001**
**Frequency (2.4 > 4 kHz)**	−**47.50**	**6**.**95**	−**6.83**	**<0.001**
Frequency (BBN > 4 KHz)	1.52	2.33	0.65	0.52
**Level × Frequency (2.4 > 4 kHz)**	**0.46**	**0**.**08**	**5.53**	**<0.001**
**Level × Frequency (BBN > 4 kHz)**	**0.11**	**0**.**05**	**2.17**	**0.03**

**Random effects**	**Variance**	***SD***		

By-participant intercepts	48.90	6.99		
Residual	15.62	3.95		

*Significant fixed effects are shown in bold.*

*The *p-*values were calculated with a Satterthwaite approximation.*

The ERB at 4 kHz is 456.46 Hz with this calculation. Next, to find the decibel level entering the filter (dB/ERB), the following equation was used, where SL is the spectrum level of the BBN.


(2) $$\frac{d\,B}{E\,R\,B}=S\,L+10\,\text{log}\,\left(E\,R\,B\right)$$

This transformation was done to make the units more comparable to those used to describe the tonal precursors; the level given is an estimate of the energy within a critical band at the signal place. Note that the off-frequency tone at 2.4 kHz does not fall within this filter; excitation from the off-frequency precursor would fall in the tail of the auditory filter, which is not captured using ERB.

Participants 1, 3, and 4 show similar estimates of gain reduction for both 4-kHz and BBN precursors when the noise level is plotted in dB/ERB units. This suggests that energy in the critical band filter dominates the elicitation of gain reduction, and that tones and noise are each able to reduce gain for a signal. P2, however, does not show similar gain reduction estimates for both 4-kHz and BBN precursors. P2’s BBN function has steeper growth than that measured with a 4-kHz tone. P2 is the same participant who did not tolerate the high pass noise presented when measuring GOM functions, indicating possible difficulty with listening tasks.

A LMM was again used to test for significant differences in slope with precursor frequency content. The statistical software and packages, criteria for included data points (data shown in Figure 6 included), dependent variable, fixed effects, reference levels, and random effects were the same as described for the analysis of Experiment 2, with the following exception. The fixed effect of precursor frequency was a categorical variable with three levels rather than two: 2.4 kHz, 4 kHz, and BBN. The model summary for the model of best fit is in Table 3. There was a significant interaction between precursor level and frequency content, such that the slope was significantly steeper in the off-frequency precursor condition compared to the on-frequency precursor condition (*p* < 0.001), as shown in Experiment 2, and the slope is significantly steeper with a BBN precursor compared to a 4-kHz precursor (*p* = 0.03). The slope of the on-frequency precursor condition fit was 0.29, the slope of the off-frequency precursor condition fit was approximately 0.46 higher, with a slope of 0.76, and the slope of the broadband noise precursor condition was approximately 0.11 higher, with a slope of 0.41 (see Table 3). Thus, the slope was far shallower in the on-frequency and broadband noise precursor conditions than in the off-frequency condition.

### Discussion

As precursor level increased, gain reduction increased with increasing precursor level at a rate less than 1 dB/dB for a BBN precursor. Estimates of gain reduction with BBN precursors increased at a significantly shallower rate than off-frequency precursors measured in Experiment 2 and at a significantly steeper rate than on-frequency precursors measured in Experiment 2. The gain reduction estimates with BBN and on-frequency precursors were closer in slope than the gain reduction estimates with BBN and off-frequency precursors (Figure 6). It was concluded that the shallow slope for both BBN and on-frequency precursors was likely related to cochlear compression.

The similarity in this study between the magnitude of gain reduction elicited by BBN and on-frequency precursors was surprising, since OAE data have suggested that noises are more robust elicitors of the ipsilateral MOCR (Lilaonitkul and Guinan, 2009a). For three of the four subjects tested, the data points for a BBN precursor are very consistent with those obtained with on-frequency tonal precursors. P2 showed a different pattern, but also had overall difficulty with the listening tasks (inferred based on inconsistency of threshold measurements). Because of this difficulty, high pass noise was removed when GOM functions were measured for P2 to obtain more consistent thresholds. The lack of consistency argues that this listener had more trouble with the task, rather than broadband noise being a stronger elicitor of gain reduction.

The estimated gain reduction measured with a BBN precursor in this study can be compared to that of other psychoacoustic studies. Yasin et al. (2014) used a different forward masking technique, in which signal and masker durations were adjusted within a 25-ms masker-signal complex to estimate the input-output function, and a precursor was presented before the masker at delays of 0-, 50-, 100-, and 200-ms. The masker was either on- or off-frequency, and the precursor was an on-frequency narrowband noise. A comparison was made between on- and off-frequency masker data to estimate cochlear gain. With this approach, they found a similar increase in cochlear gain reduction with precursor level; they reported a slope of 0.33 for the 0-ms delay condition, which is similar to that measured in this experiment for on-frequency and BBN precursors. Maximum gain reduction was approximately 25 dB (Yasin et al., 2014), consistent with the current results. In another study using pink-noise precursors, approximately 10 dB of gain reduction was estimated with a 60 dB SPL overall precursor level with a 50-ms duration, again consistent with the present results with a BBN (DeRoy Milvae and Strickland, 2018). The estimated gain reduction in this study and Yasin et al. (2014) is largely consistent with physiological measures (Russell and Murugasu, 1997; Cooper and Guinan, 2006). Maximum gain reduction of 25 dB is larger than the 15–20 dB of maximum gain reduction measured physiologically (Russell and Murugasu, 1997; Cooper and Guinan, 2006). A difference between the psychoacoustic measures and physiological measure is that cochlear gain reduction was evoked by sound psychoacoustically, but by electrical pulses physiologically (Dolan et al., 1997; Russell and Murugasu, 1997). Therefore, the psychoacoustic estimates of gain reduction confirm that the decibel levels measured with electrical stimulation are plausible in a natural listening situation. It is possible that greater gain reduction emerges psychoacoustically due to differences in the stimulation mode, differences across species, or forward masking contributions unrelated to cochlear gain reduction at high levels.

## General Discussion

In Experiment 1, GOM functions were measured with and without preceding sound to obtain an estimate of each participant’s cochlear input-output function at full gain and with decreased gain (Figure 3). In addition, the theory that the masking provided by the precursor is due to decreased cochlear gain was tested. Equally effective on- and off-frequency maskers were found, and the same precursor was added to each condition. Additivity of forward masking predicts that this addition of the precursors would lead to a similar shift in signal threshold, regardless of the masker frequency. However, there was a larger shift in signal threshold for the off-frequency masker condition (Figure 4). This is consistent with a gain reduction hypothesis to explain the additional forward masking. In Experiments 2 and 3, gain reduction was estimated for a range of precursor levels (Figures 5, 6). On-frequency, off-frequency, and BBN precursors were used. Increases in estimated gain reduction with increased precursor level varied in slope, with a shallower slope for on-frequency and BBN precursors than off-frequency precursors (Tables 2, 3). It is possible that the shallow slopes seen with the on-frequency and BBN precursors were due to cochlear compression, and that elicitation of gain reduction with level reflects growth of excitation within a cochlear channel at or near the signal frequency.

### Theories of Forward Masking

In Experiment 1, when on- and off-frequency maskers were matched in effectiveness, producing very similar signal thresholds, the addition of an identical precursor caused a divergence in signal threshold. This result is more consistent with a gain reduction theory of forward masking than additivity of masking, and supports the idea that the threshold shift with precursors used to measure GOM functions was due to cochlear gain reduction.

The delay between the onset of the precursor and signal was long enough for gain reduction to occur at the signal place (Backus and Guinan, 2006). Since gain reduction is frequency-specific for ipsilateral tone elicitors (Lilaonitkul and Guinan, 2009a), the on-frequency precursor would elicit its strongest gain reduction at or near the 4-kHz place in the cochlea, where the subjects are assumed to be listening. Because the off-frequency masker is almost an octave lower than 4 kHz, it would be processed linearly at the 4-kHz place (Ruggero et al., 1997; Cooper and Guinan, 2006). This linear processing means that the off-frequency masker has no gain to be turned down at the signal place. However, the 4-kHz masker does have gain that can be turned down at the 4-kHz place due to the presence of the precursor. This differential impact of cochlear gain reduction on the two maskers leads to reduced gain for the 4-kHz masker and no change for the 2.4-kHz masker (Kawase et al., 2000). Therefore, the 2.4-kHz masker is then more effective than the 4-kHz masker, since they were matched in effectiveness in a condition without preceding sound. This leads to higher signal thresholds for the off-frequency masker condition.

### Tonal and Noise Precursor Data Support Frequency Specificity

To produce the same amount of gain reduction, the precursor level had to be higher when it was off-frequency than when it was on-frequency. This is consistent with OAE data (Lilaonitkul and Guinan, 2009b) and psychoacoustic data (Jennings and Strickland, 2010) showing frequency selectivity for the precursor. The precursor duration used in the present study is quite short, 50 ms, because this was found to be the most effective duration for an on-frequency precursor (Roverud and Strickland, 2014). For an off-frequency precursor, however, a longer duration would likely have produced more gain reduction. Roverud and Strickland (2014) modeled this as the on-frequency precursor being reduced by the gain reduction it produced, while the off-frequency precursor produced gain reduction but was not affected itself. This could be part of the reason that both OAE data and psychoacoustic data (Drga et al., 2016) show that when equal-level, long-duration elicitors are used, a broad range of elicitor frequencies are effective.

The similarity between gain reduction measured with on-frequency and BBN precursors leads to two conclusions. The first is that gain reduction masking is dominated by contributing energy in the critical bandwidth. In this way, the on-frequency tone and BBN with equal decibel level within the critical bandwidth produce similar thresholds. The second is that on-frequency tones and BBN are almost equally effective elicitors of gain reduction for the elicitor duration used here. This differs from OAE data that have suggested that noises are more robust elicitors of the ipsilateral MOCR (Lilaonitkul and Guinan, 2009a). It is possible that broadband stimuli lead to a larger change in otoacoustic emissions unrelated to greater elicitation of cochlear gain reduction. Larger bandwidth effects are also seen with contralateral elicitation (Maison et al., 2000; Lilaonitkul and Guinan, 2009a) than with ipsilateral elicitation (Lilaonitkul and Guinan, 2009a), so it may be that contralateral elicitation of cochlear gain reduction does integrate across cochlear place, but that this is not the case for ipsilateral elicitation. It is also possible that the effects depend on the signal or probe frequency; larger bandwidth effects were seen at lower frequencies using SFOAEs (Lilaonitkul and Guinan, 2009a). This could be explored in future experiments with behavioral measures.

The slope of increased gain reduction with increased BBN precursor level has implications for gain reduction research. The shallow slope means that small changes in input level of elicitor of the MOCR do not lead to large changes in cochlear gain reduction. This means that studies with differing elicitor levels can more easily be compared; differences in input level are smaller at the output of the system. However, for off-frequency elicitors, gain reduction increases with a faster rate as level is increased. This may be important in real-world situations where the noise may be low frequency.

### Demonstrated Impact of Gain Reduction on Perception

This experiment demonstrated that gain reduction, measured psychophysically, grows with level and this growth varies in slope depending on the elicitor used. The authors interpret the data as supporting that the excitation at the signal place, regardless of frequency, determines the amount of gain reduction. Different frequencies will differ in the compression applied to the input, affecting the slope of estimated gain reduction with increasing level. The similar shallow slope for increased gain reduction as a function of level with on-frequency and BBN precursors suggests that ipsilateral BBN elicitors are similarly effective to tonal elicitors, contrary to findings with SFOAE measurements (Lilaonitkul and Guinan, 2009a). The MOCR provides a mechanism for the peripheral auditory system to adaptively vary cochlear gain. This study supported that the amount of gain reduction increases with increasing level of the auditory environment, which may help the auditory system to remain sensitive to new information over the wide range of levels that we can hear.

## Data Availability Statement

The raw data supporting the conclusions of this article will be made available by the authors, without undue reservation.

## Ethics Statement

The studies involving human participants were reviewed and approved by the Institutional Review Board at Purdue University. The participants provided their written informed consent to participate in this study.

## Author Contributions

KDM was involved in research design, collected data, analyzed the data, and wrote and edited the manuscript. ES guided the research design and wrote and edited the manuscript. Both authors made significant contributions to warrant authorship and approved the final version for submission.

## Conflict of Interest

The authors declare that the research was conducted in the absence of any commercial or financial relationships that could be construed as a potential conflict of interest.

## Publisher’s Note

All claims expressed in this article are solely those of the authors and do not necessarily represent those of their affiliated organizations, or those of the publisher, the editors and the reviewers. Any product that may be evaluated in this article, or claim that may be made by its manufacturer, is not guaranteed or endorsed by the publisher.
